# Supplementary material for: NNMT Loss Drives Cancer Progression by enhancing SAM availability for mTORC1 Signaling and Chromatin Methylation
Source: bioRxiv. 2026 Jul 1:2026.06.30.734970. Preprint. [Version 1] doi: 10.64898/2026.06.30.734970 (PMC13430563; doi:10.64898/2026.06.30.734970)
Supplement: Supplementary file 1 [file NIHPP2026.06.30.734970v1-supplement-1.pdf]

bioRxiv preprint doi: https://doi.org/10.64898/2026.06.30.734970; this version posted July 1, 2026. The copyright holder for this preprint (which was not certified by peer review) is the author/funder, who has granted bioRxiv a license to display the preprint in perpetuity. It is made available under a CC-BY 4.0 International license.

Supplementary Figure 1

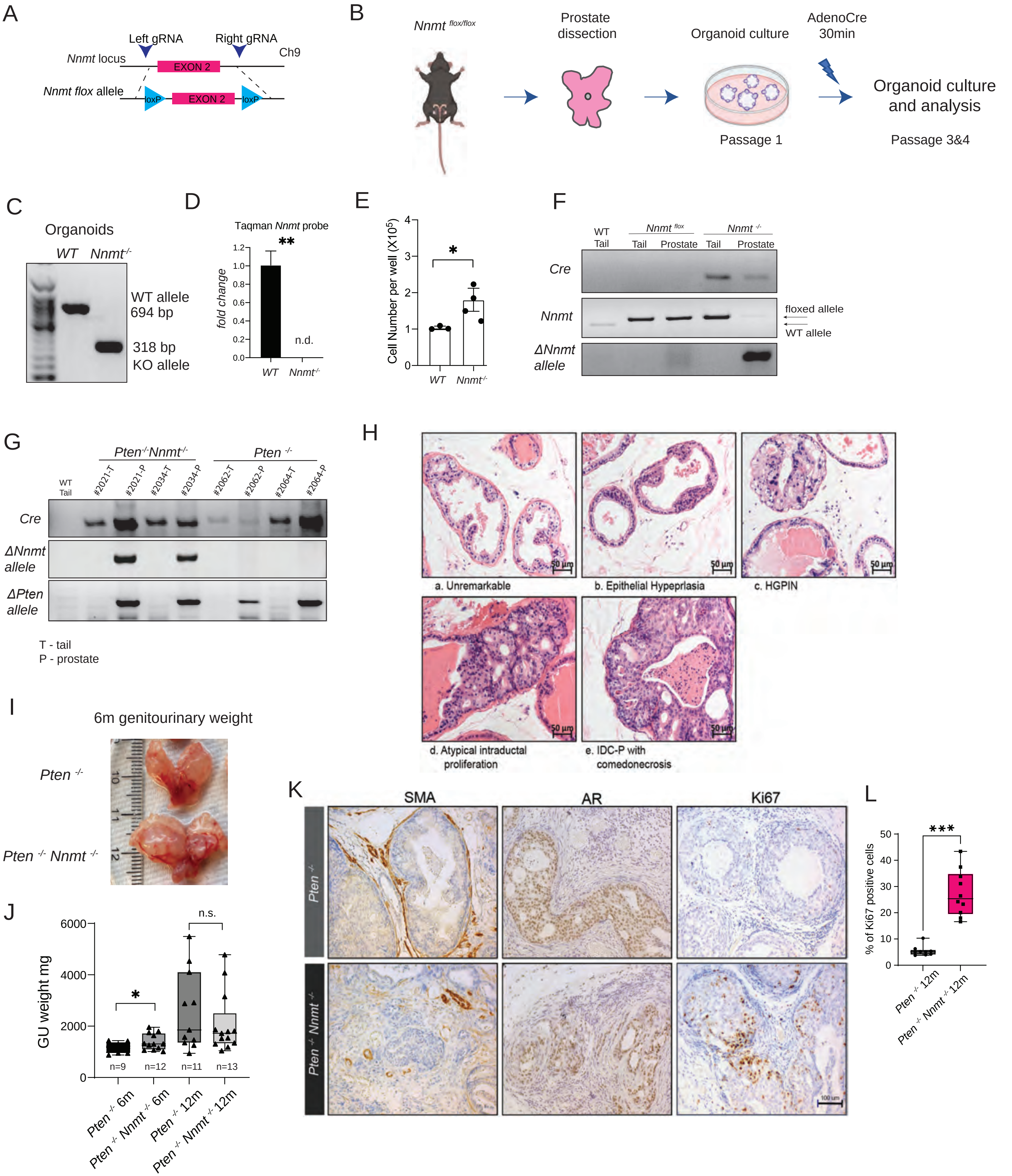

bioRxiv preprint doi: https://doi.org/10.64898/2026.06.30.734970; this version posted July 1, 2026. The copyright holder for this preprint (which was not certified by peer review) is the author/funder, who has granted bioRxiv a license to display the preprint in perpetuity. It is made available under a CC-BY 4.0 International license.

Figure S1. *Nnmt* deletion promotes prostate cancer progression in mice.

(A) Construction strategy for CRISPR-Cas9-mediated knock-in of flox sites in *Nnmt* allele.

(B) *In vitro* knockout of *Nnmt* in prostate epithelial organoids increases proliferation. Schematic of organoid culture and AdenoCre-mediated *Nnmt* knockout *in vitro.*

(C) PCR amplification showing *Nnmt* knockout allele using genomic DNA from the indicated organoids.

(D) TaqMan qRT-PCR confirming NNMT loss in *Nnmt*$^{-/-}$ organoids.

(E) Quantification of cell numbers in organoid culture experiments using the indicated cells as shown in Figure 1B.

(F) Prostate-specific *Nnmt* knockout in mice. PCR amplification showing *ΔNnmt* fragment in *Nnmt*$^{-/-}$ mouse prostate. *Nnmt*^{flox} and tail DNA were used as negative controls.

(G) Prostate-specific *Nnmt* and *Pten* double knockout in mice. PCR amplification showing ΔNnmt and ΔPten fragments in *Nnmt*$^{-/-}$*Pten*$^{-/-}$ mouse prostate. *Pten*$^{-/-}$ and tail DNA were used as negative controls.

(H) Pathological categorization of the identified lesions in knockout mice. Representative H&E images of lesions.

(I-J) *Pten*$^{-/-}$*Nnmt*$^{-/-}$ mice develop larger prostates at 6 months. (I) Gross image of representative prostate and seminal vesicle (J) Quantification *of* genitourinary weights at 6 and 12 months.

(K) Representative IHC images of Smooth muscle Actin (SMA), AR and Ki67 staining showing infiltrating carcinoma developed in *Pten*$^{-/-}$*Nnmt*$^{-/-}$ prostate at 12 months.

(L) Quantification of Ki67 staining.

Statistical significance was assessed using unpaired two-sided Student's t-test (*P < 0.05, **P < 0.01, ***P < 0.001 n.s. non-significant).

bioRxiv preprint doi: https://doi.org/10.64898/2026.06.30.734970; this version posted July 1, 2026. The copyright holder for this preprint (which was not certified by peer review) is the author/funder, who has granted bioRxiv a license to display the preprint in perpetuity. It is made available under a CC-BY 4.0 International license.

Supplementary Figure 2

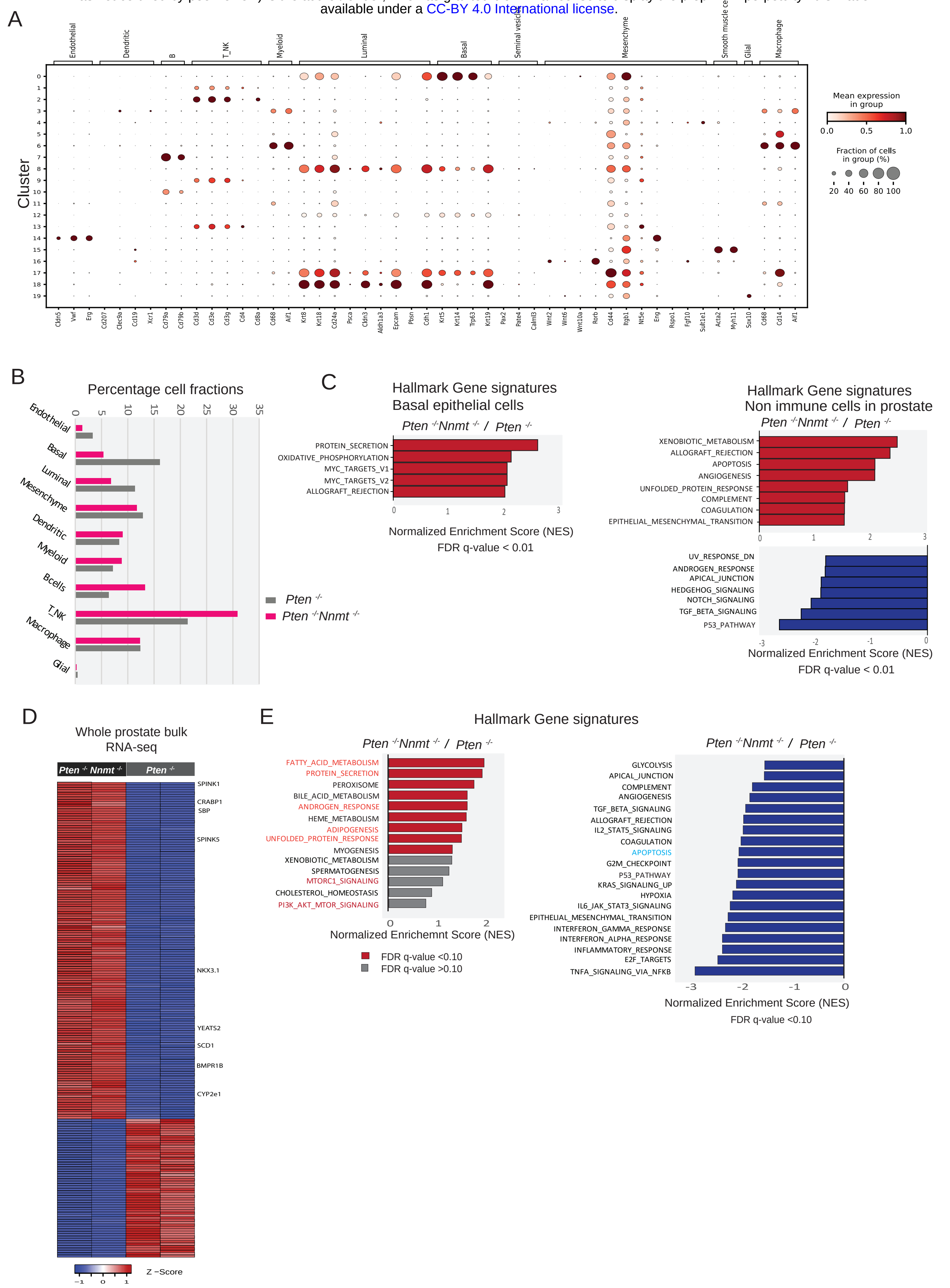

bioRxiv preprint doi: https://doi.org/10.64898/2026.06.30.734970; this version posted July 1, 2026. The copyright holder for this preprint (which was not certified by peer review) is the author/funder, who has granted bioRxiv a license to display the preprint in perpetuity. It is made available under a CC-BY 4.0 International license.

Figure S2. NNMT loss is associated with transcriptional changes

(A) Bubble plot of unique marker genes from different cell types identified through scRNA-seq in *Pten*$^{-/-}$*Nnmt*$^{-/-}$ and *Pten*$^{-/-}$ mouse prostate.

(B) Distribution fraction of different cell types in *Pten*$^{-/-}$*Nnmt*$^{-/-}$ and *Pten*$^{-/-}$ prostate.

(C) List of significant Hallmark gene signatures in basal epithelial and non-immune cells from *Pten*$^{-/-}$*Nnmt*$^{-/-}$ versus *Pten*$^{-/-}$ prostate. Note the absence of mTOR and AR gene signatures in these cell population unlike luminal epithelium shown in Figure 2.

(D) Heatmap of differentially expressed genes derived from RNA-seq of *Pten*$^{-/-}$*Nnmt*$^{-/-}$ and *Pten*$^{-/-}$ whole prostate tissue from mice fed with methionine-restricted diet.

(E) Enriched and depleted Hallmark gene signatures in *Pten*$^{-/-}$*Nnmt*$^{-/-}$ prostates from mice fed with methionine-restricted diet.

bioRxiv preprint doi: https://doi.org/10.64898/2026.06.30.734970; this version posted July 1, 2026. The copyright holder for this preprint (which was not certified by peer review) is the author/funder, who has granted bioRxiv a license to display the preprint in perpetuity. It is made available under a CC-BY 4.0 International license.

Supplementary Figure 3

A

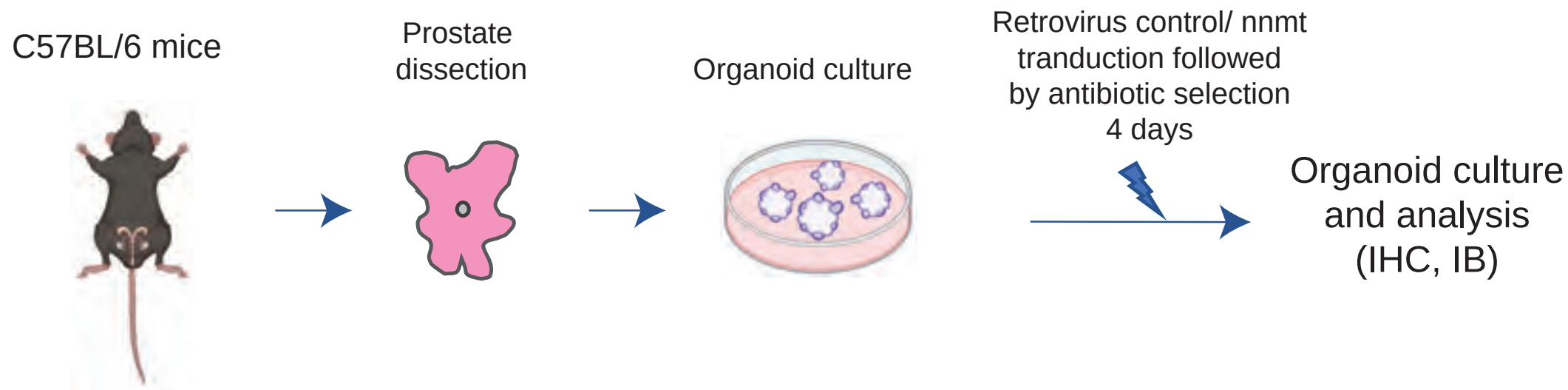

B

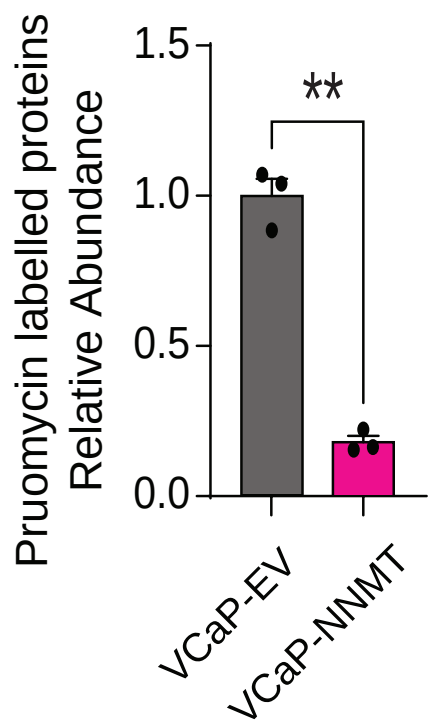

bioRxiv preprint doi: https://doi.org/10.64898/2026.06.30.734970; this version posted July 1, 2026. The copyright holder for this preprint (which was not certified by peer review) is the author/funder, who has granted bioRxiv a license to display the preprint in perpetuity. It is made available under a CC-BY 4.0 International license.

Figure S3. NNMT modulates mTORC1 signaling and protein translation

(A) Schematic of prostate organoid culture and retrovirus based NNMT overexpression.

(B) Quantification of the immunoblot signal from Figure 3H. Statistical significance was assessed using a paired two-sided Student's t-test (**$P < 0.01$).

bioRxiv preprint doi: https://doi.org/10.64898/2026.06.30.734970; this version posted July 1, 2026. The copyright holder for this preprint (which was not certified by peer review) is the author/funder, who has granted bioRxiv a license to display the preprint in perpetuity. It is made available under a CC-BY 4.0 International license.

Supplementary Figure 4

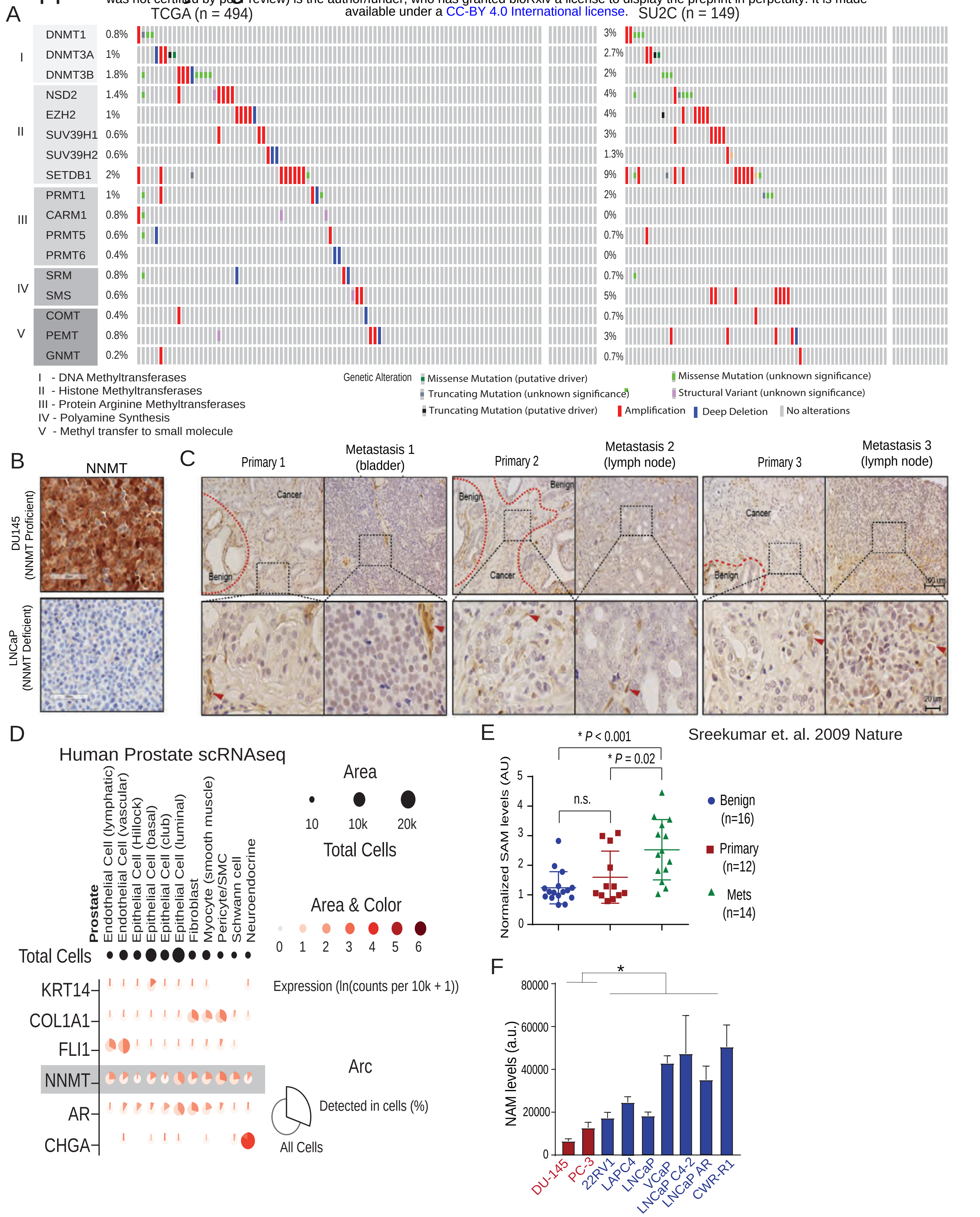

bioRxiv preprint doi: https://doi.org/10.64898/2026.06.30.734970; this version posted July 1, 2026. The copyright holder for this preprint (which was not certified by peer review) is the author/funder, who has granted bioRxiv a license to display the preprint in perpetuity. It is made available under a CC-BY 4.0 International license.

Figure S4. Recurrent genomic deletion and silencing of NNMT in human prostate cancer

(A) Oncoprint from CBioPortal showing genetic alterations in major SAM-consuming enzymes in primary (TCGA) and metastatic (SU2C) prostate cancer datasets.

(B) NNMT antibody validation DU145 and LNCaP cells.

(C) NNMT expression is lost in human PCa. IHC images showing NNMT expression in matched primary and metastatic human PCa tissue samples. The lower panels are magnified selected regions and red arrows indicate NNMT-positive non-cancerous cells.

(D) NNMT expression in normal human prostate. The bubble plot illustrating the expression levels of NNMT alongside other cell type-specific genes across various cell types within the normal prostate, as derived from GTEx Portal.

(E) Elevated SAM levels in prostate cancer metastases. LC-MS quantification of SAM levels in benign, localized PCa, and metastatic castration-resistant prostate cancer (CRPC) tissues. The plot was generated from data within the published metabolome datasets (PMID: 19212411).

(F) LC-HRMS-based quantification of nicotinamide (NAM) levels in PCa cell lines.

bioRxiv preprint doi: https://doi.org/10.64898/2026.06.30.734970; this version posted July 1, 2026. The copyright holder for this preprint (which was not certified by peer review) is the author/funder, who has granted bioRxiv a license to display the preprint in perpetuity. It is made available under a CC-BY 4.0 International license.

Supplementary Figure 5

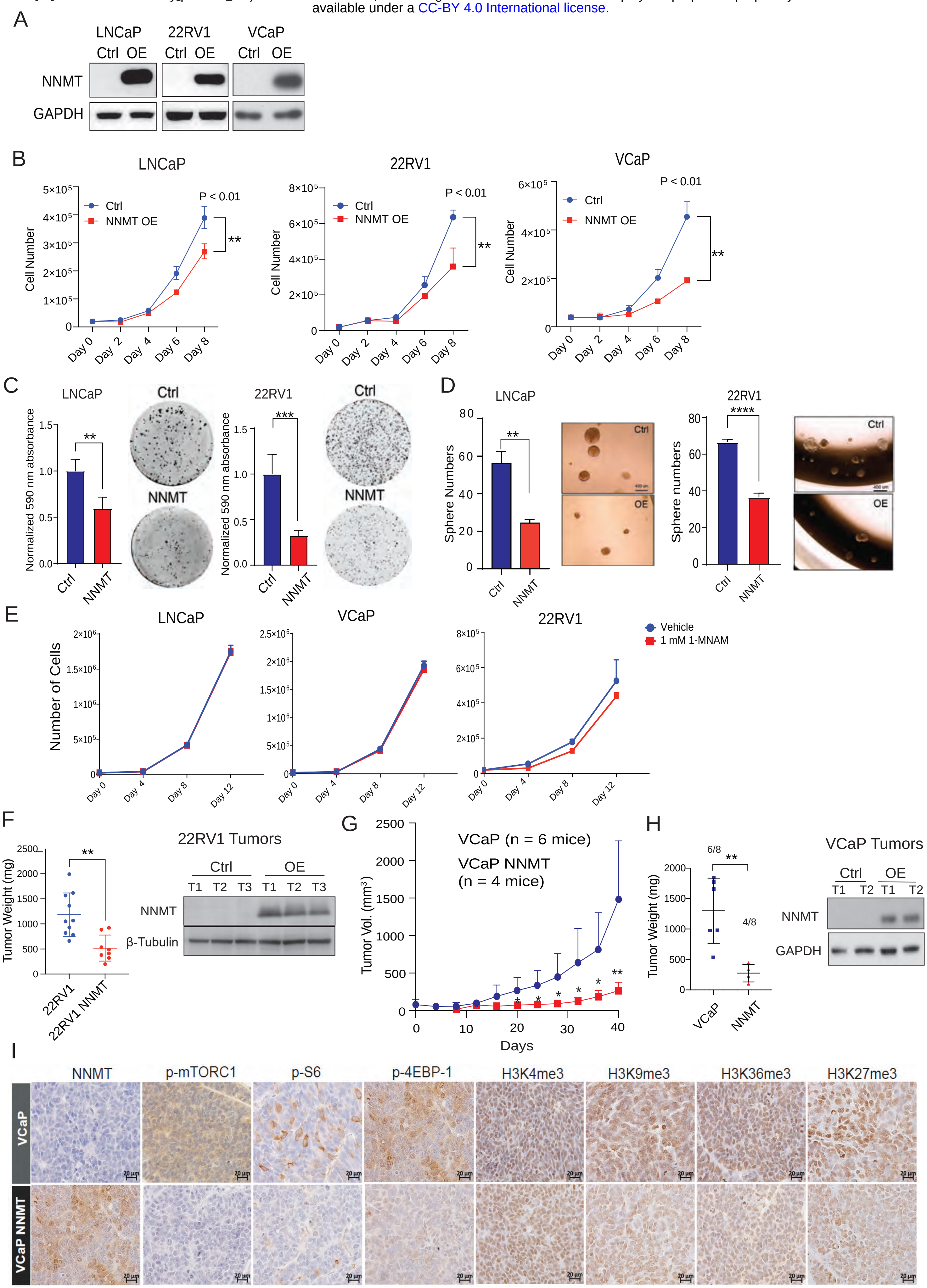

bioRxiv preprint doi: https://doi.org/10.64898/2026.06.30.734970; this version posted July 1, 2026. The copyright holder for this preprint (which was not certified by peer review) is the author/funder, who has granted bioRxiv a license to display the preprint in perpetuity. It is made available under a CC-BY 4.0 International license.

Figure S5. NNMT overexpression suppresses prostate cancer growth *in vitro* and *in vivo*

(A) NNMT overexpression models in PCa cells. Immunoblots confirm NNMT expression in engineered cells.

(B-D) NNMT overexpression suppress PCa growth *in vitro*. (B) Cell proliferation rates over time. (C-D) Quantifications and representative images of colony (C) and sphere (D) formation assays. Cells were grown in regular media for three assays.

(E) 1-MNAM has no effect on prostate cancer cell proliferation. Growth rates of LNCaP, VCaP, and 22RV1 cells cultured with 1mM 1-MNAM or vehicle over time.

(F-H) NNMT overexpression suppresses AR-positive PCa xenograft growth in mice. (F) Endpoint 22RV1 tumor weight from main Figure 4F. Immunoblots confirming NNMT overexpression in xenograft lysates. (G,H) As in F with VCaP and VCaP NNMT overexpression model. (I) Reduced mTOR signaling and histone methylation in NNMT-overexpressing VCaP xenografts. Representative IHC images of the phospho-proteins in mTORC1 pathway and indicated histone methylation marks in control and NNMT-overexpressing VCaP tumor xenografts.

Statistical significance was assessed using an unpaired two-sided Student's t-test (*$P < 0.05$, **$P < 0.01$, ***$P < 0.001$ ****$P < 0.0001$).

bioRxiv preprint doi: https://doi.org/10.64898/2026.06.30.734970; this version posted July 1, 2026. The copyright holder for this preprint (which was not certified by peer review) is the author/funder, who has granted bioRxiv a license to display the preprint in perpetuity. It is made available under a CC-BY 4.0 International license.

Supplementary Figure 6

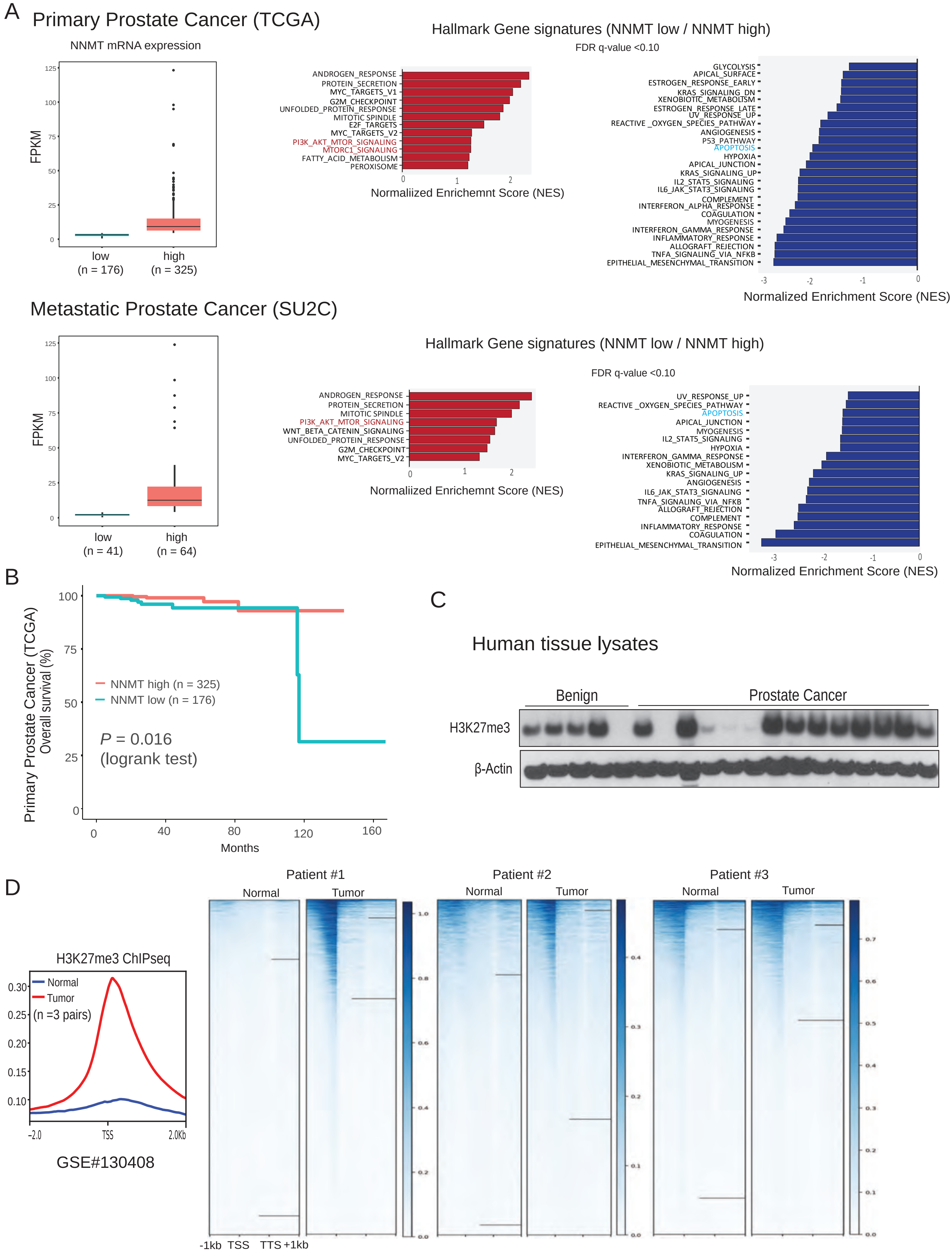

bioRxiv preprint doi: https://doi.org/10.64898/2026.06.30.734970; this version posted July 1, 2026. The copyright holder for this preprint (which was not certified by peer review) is the author/funder, who has granted bioRxiv a license to display the preprint in perpetuity. It is made available under a CC-BY 4.0 International license.

Figure S6. Altered gene expression pathways and histone methylation in human PCa

(A) Top-ranked significantly enriched and depleted Hallmark gene signatures in NNMT-low versus NNMT-high tumors from TCGA and SU2C prostate cancer datasets. Box plots show NNMT expression in low and high cases.

(B) Kaplan-Meier survival curves for NNMT low and high cases in TCGA dataset.

(C) Immunoblots of histone H3K27me3 marks in human PCa tumor and benign prostate tissue lysates. b-Actin served as loading control.

(D) *left*, H3K27me3 ChIP-seq in matched normal and PCa, reanalyzed from GSE1304408. *Right*, heatmap representation across +/- 2Kb TSS and TTS.

bioRxiv preprint doi: https://doi.org/10.64898/2026.06.30.734970; this version posted July 1, 2026. The copyright holder for this preprint (which was not certified by peer review) is the author/funder, who has granted bioRxiv a license to display the preprint in perpetuity. It is made available under a CC-BY 4.0 International license.

Supplemental Figure 7

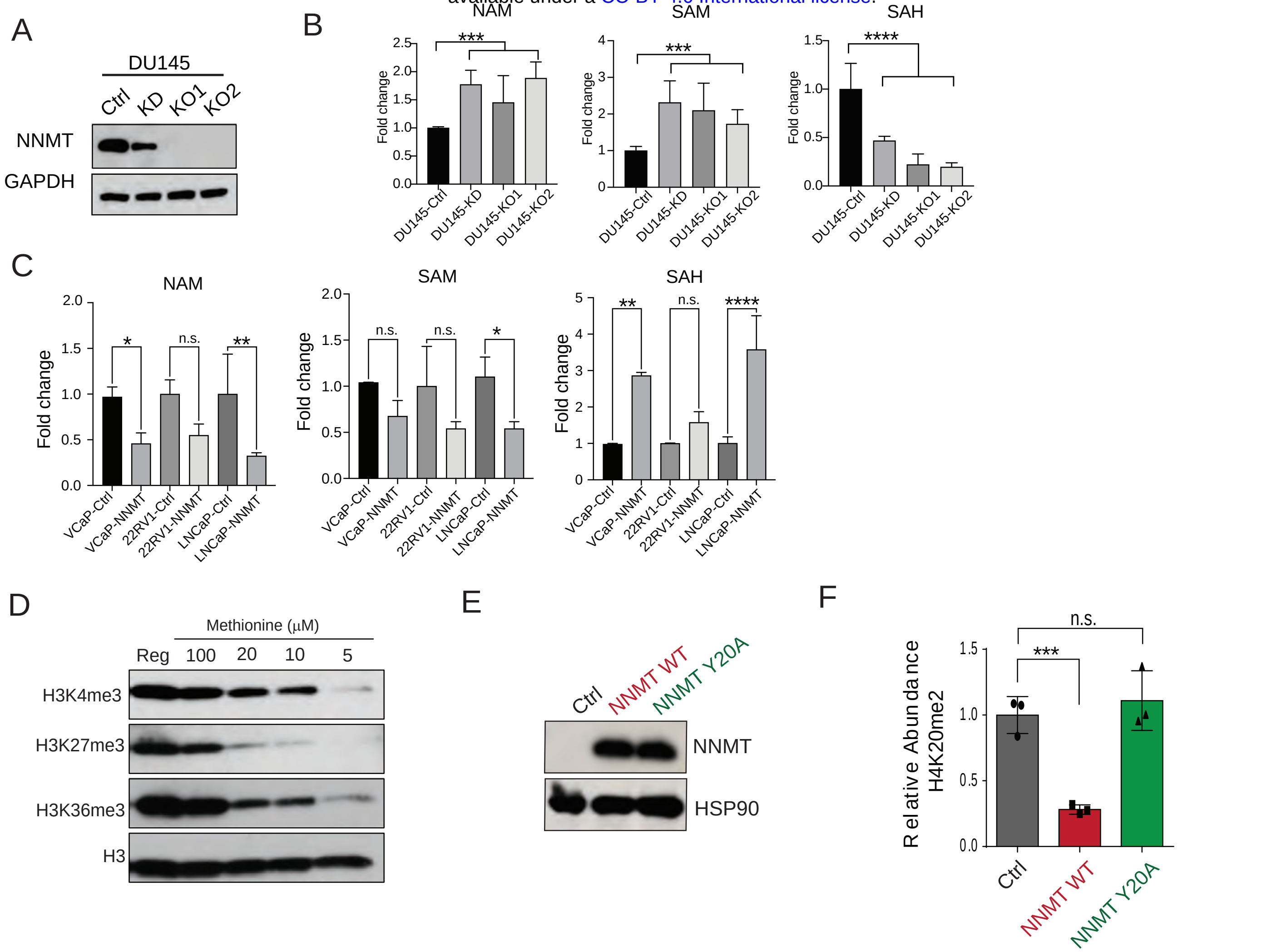

bioRxiv preprint doi: https://doi.org/10.64898/2026.06.30.734970; this version posted July 1, 2026. The copyright holder for this preprint (which was not certified by peer review) is the author/funder, who has granted bioRxiv a license to display the preprint in perpetuity. It is made available under a CC-BY 4.0 International license.

Figure S7. NNMT catalytic activity regulates cellular SAM levels, while MNAM does not influence prostate cancer cell growth

(A) Establishment of NNMT knockdown and knockout models. Immunoblots confirm NNMT depletion.

(B-C) NNMT depletes cellular SAM levels. LC-MS analysis shows reciprocal changes in NAM, SAM, and SAH levels in NNMT KO and overexpressing prostate cancer cells.

(D) DU145 cells grown in the indicated methionine concentration for 12-days. Histone extracts were probed with the indicated antibody.

(E) Immunoblots of lentivirus-based wildtype NNMT or the NNMT Y20A catalytic-dead mutant expression in LNCaP cells.

(F) Quantification of H4K20me2 immunoblot shown in Figure 5H.

Statistical significance was assessed using an unpaired two-sided Student’s t-test (*$P < 0.05$, **$P < 0.01$, ***$P < 0.001$ ****$P < 0.0001$, n.s. non-significant).

bioRxiv preprint doi: https://doi.org/10.64898/2026.06.30.734970; this version posted July 1, 2026. The copyright holder for this preprint (which was not certified by peer review) is the author/funder, who has granted bioRxiv a license to display the preprint in perpetuity. It is made available under a CC-BY 4.0 International license.

Supplemental Figure 8

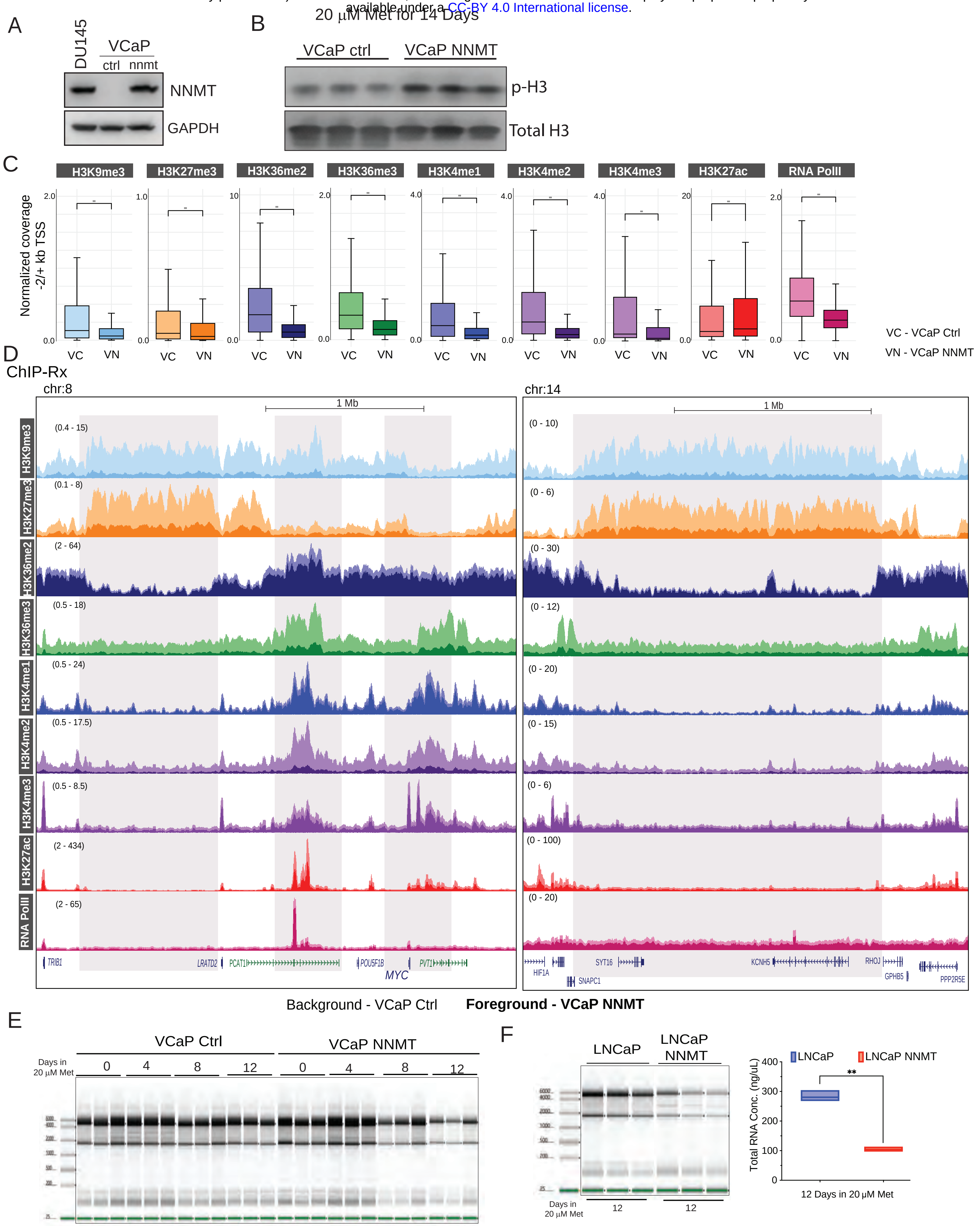

bioRxiv preprint doi: https://doi.org/10.64898/2026.06.30.734970; this version posted July 1, 2026. The copyright holder for this preprint (which was not certified by peer review) is the author/funder, who has granted bioRxiv a license to display the preprint in perpetuity. It is made available under a CC-BY 4.0 International license.

Figure S8. NNMT depletes histone methylation genome-wide and suppresses transcription in prostate cancer cells

(A) Immunoblots of NNMT in DU-145, VCaP, and VCaP NNMT overexpressing cells.

(B) Immunoblot of phospho-H3, a surrogate for mitotic cells, in VCaP control and NNMT-overexpressing cells, grown in physiological concentration of methionine for 14-days.

(C-D) NNMT expression decreases histone methylation. (C) Histone methylation and acetylation marks and RNA PolII at TSS (+/- 2kb) (D) Genome browser view of transcriptionally active (*MYC* locus) and silenced (*KCNH5* locus) regions. The background lighter shade signal is from VCaP control, and the darker shade signal in the foreground is from VCaP NNMT overexpressing cells. The gray box indicates mutually exclusive hetero- and euchromatin regions. Data normalized with drosophila chromatin spike-in controls (ChIP-Rx).

(E-F) NNMT expression suppresses the transcription rate in PCa cells. (E) TapeStation gel images for RNA from VCaP control and NNMT overexpressing VCaP cells grown under physiological methionine conditions – as in Fig.5I. (F) TapeStation gel image with total RNA and its concentrations from NNMT-overexpressing LNCaP cells.

Statistical significance was assessed using an unpaired two-sided Student’s t-test, $^{**}P < 0.01$.

bioRxiv preprint doi: https://doi.org/10.64898/2026.06.30.734970; this version posted July 1, 2026. The copyright holder for this preprint (which was not certified by peer review) is the author/funder, who has granted bioRxiv a license to display the preprint in perpetuity. It is made available under aCC-BY 4.0 International license.

Supplementary Figure 9

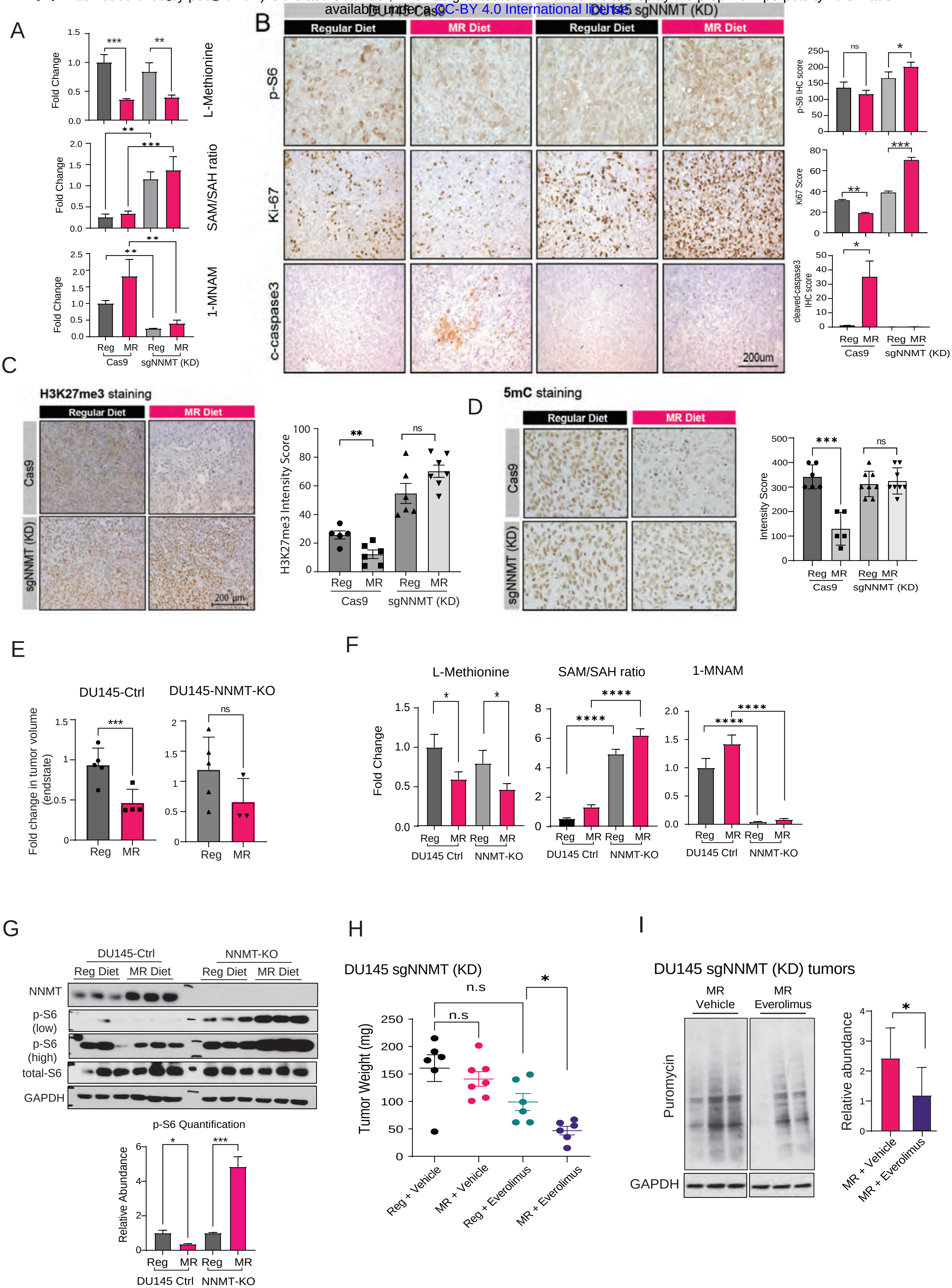

bioRxiv preprint doi: https://doi.org/10.64898/2026.06.30.734970; this version posted July 1, 2026. The copyright holder for this preprint (which was not certified by peer review) is the author/funder, who has granted bioRxiv a license to display the preprint in perpetuity. It is made available under a CC-BY 4.0 International license.

Figure S9: NNMT deficiency creates therapeutic vulnerability to mTOR inhibitor when combined with methionine restricted diet.

(A) MR-diet reduces methionine levels in xenograft tumors. LC-MS quantification of methionine, SAM/SAH ratio, and 1-MNAM in DU145 cas9 control and DU145 NNMT KD tumor lysates.

(B) MR-diet induces p-S6 and mitotic activity in the NNMT KD tumors. Representative IHC images and quantifications.

(C-D) Compared to NNMT-proficient controls NNMT KD tumors show no reduction in H3K27me3 and 5-methylated Cytosine (5mC) levels under MR-diet. Representative IHC images and quantifications.

(E-G) NNMT-knockout DU145 tumors are refractory to MR-diet through activation of mTOR signaling. (E) Endpoint tumor volume under MR (n = 4) or regular diet (n=5). (F) LC-MS quantification of SAM metabolites. (G) Immunoblots and quantifications of p-S6.

(H) MR-diet synergizes with Everolimus (mTOR inhibitor) to suppress DU145 NNMT-KD tumors. Endpoint tumor weights in DU145 NNMT knockdown xenografts as shown in Figure 6F.

(I) *In vivo* SUnSET assay shows reduced protein synthesis by MR and Everolimus treatment in NNMT-KD DU145 xenografts. *Left,* immunoblots of puromycin using the indicated xenograft lysates, each lane represents an independent tumor. *Right,* Quantifications.

Statistical significance was assessed using an unpaired two-sided Student's t-test (*$P < 0.05$, **$P < 0.01$, ***$P < 0.001$ ***$P < 0.0001$, n.s. non-significant).

bioRxiv preprint doi: https://doi.org/10.64898/2026.06.30.734970; this version posted July 1, 2026. The copyright holder for this preprint (which was not certified by peer review) is the author/funder, who has granted bioRxiv a license to display the preprint in perpetuity. It is made available under a CC-BY 4.0 International license.

Supplementary Figure 10

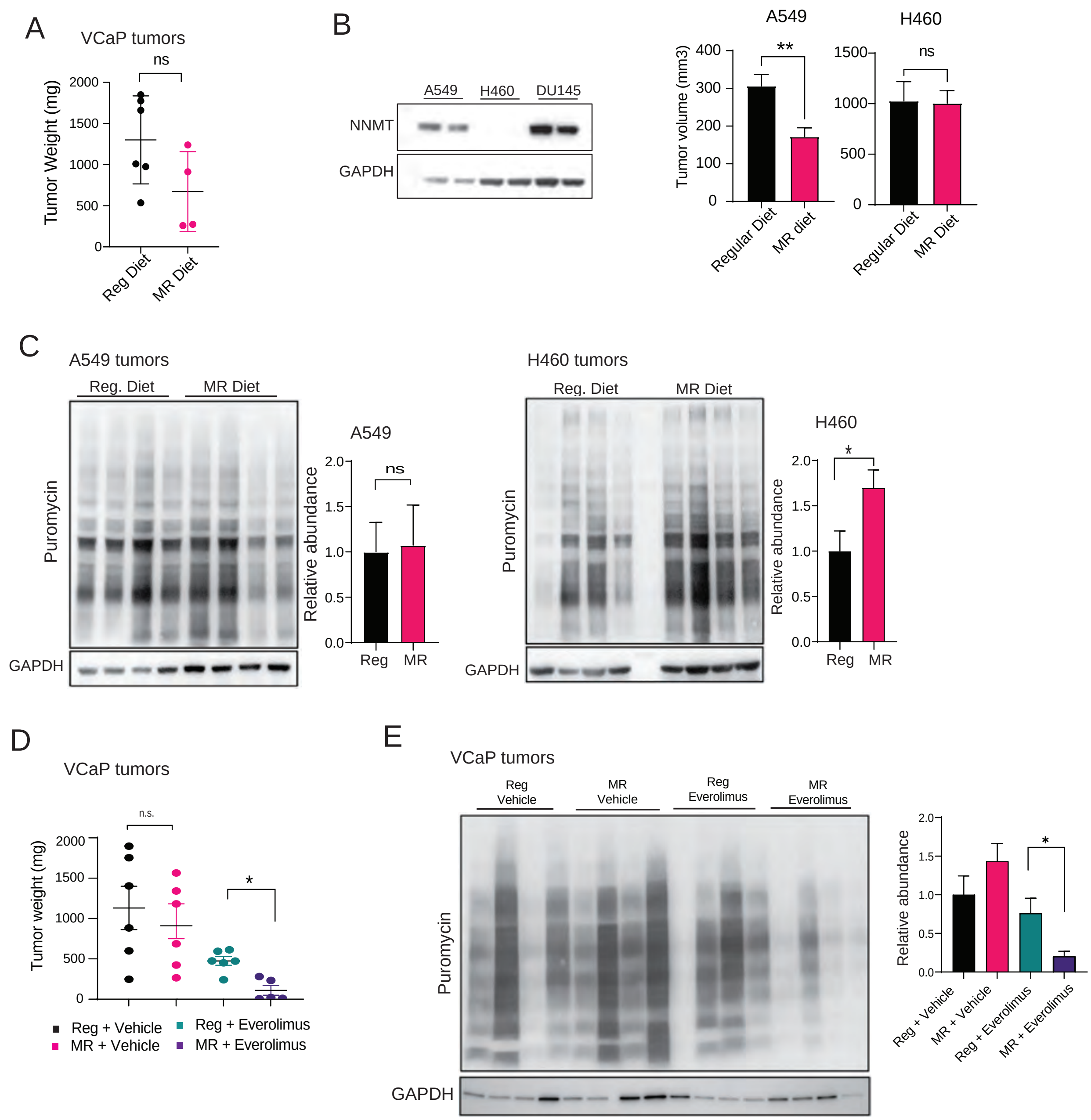

bioRxiv preprint doi: https://doi.org/10.64898/2026.06.30.734970; this version posted July 1, 2026. The copyright holder for this preprint (which was not certified by peer review) is the author/funder, who has granted bioRxiv a license to display the preprint in perpetuity. It is made available under a CC-BY 4.0 International license.

Figure S10: NNMT deficiency creates therapeutic vulnerability to mTOR inhibitor when combined with methionine restricted diet.

(A) NNMT-deficient VCaP xenografts are refractory to MR-diet. Endpoint tumor weight of a VCaP xenograft assay in mice fed with regular (n = 6) or MR-diet (n = 4).

(B-C) NNMT-deficient human lung xenografts are refractory to MR-diet (B) *Left*, immunoblots of NNMT in human lung cancer cell lines: A549 and H460 and PCa cell line DU145. *Right*, endpoint tumor volume in NNMT-proficient A549 and NNMT-deficient H460 xenograft assays in mice fed with regular or MR-diet. (C) Immunoblots of puromycin using the indicated xenograft lysates, each lane represents an independent tumor. *Right*, Quantifications

(D-E) MR-diet combined with Everolimus suppresses VCaP xenograft growth. Endpoint VCaP xenograft tumor weights and immunoblots of puromycin using the indicated xenograft lysates, each lane represents an independent tumor. *Right*, Quantifications

Statistical significance was assessed using an unpaired two-sided Student's t-test (*$P < 0.05$, **$P < 0.01$, n.s. non-significant).
